# Deep Convolutional Backbone Comparison for Automated PET Image Quality Assessment

**DOI:** 10.1109/TRPMS.2024.3436697

**Published:** 2024-11

**Authors:** Jessica B. Hopson, Anthime Flaus, Colm J. McGinnity, Radhouene Neji, Andrew J. Reader, Alexander Hammers

**Affiliations:** Department of Biomedical Engineering, King’s College London; King’s College London & Guy’s and St Thomas’ PET Centre, King’s College London; King’s College London & Guy’s and St Thomas’ PET Centre, King’s College London; Department of Biomedical Engineering, King’s College London; Siemens Healthcare Limited; Department of Biomedical Engineering, King’s College London; King’s College London & Guy’s and St Thomas’ PET Centre, King’s College London

**Keywords:** Convolutional neural networks, Deep learning, Image quality, Image reconstruction, Transfer learning

## Abstract

Pretraining deep convolutional network mappings using natural images helps with medical imaging analysis tasks; this is important given the limited number of clinically-annotated medical images. Many two-dimensional pretrained backbone networks, however, are currently available. This work compared 18 different backbones from 5 architecture groups (pretrained on ImageNet) for the task of assessing [^18^F]FDG brain Positron Emission Transmission (PET) image quality (reconstructed at seven simulated doses), based on three clinical image quality metrics (global quality rating, pattern recognition, and diagnostic confidence). Using two-dimensional randomly sampled patches, up to eight patients (at three dose levels each) were used for training, with three separate patient datasets used for testing. Each backbone was trained five times with the same training and validation sets, and with six cross-folds. Training only the final fully connected layer (with ~6,000–20,000 trainable parameters) achieved a test mean-absolute-error of ~0.5 (which was within the intrinsic uncertainty of clinical scoring). To compare “classical” and over-parameterized regimes, the pretrained weights of the last 40% of the network layers were then unfrozen. The mean-absolute-error fell below 0.5 for 14 out of the 18 backbones assessed, including two that previously failed to train. Generally, backbones with residual units (e.g. DenseNets and ResNetV2s), were suited to this task, in terms of achieving the lowest mean-absolute-error at test time (~0.45 – 0.5). This proof-of-concept study shows that over-parameterization may also be important for automated PET image quality assessments.

## I Introduction

Transfer-learning is defined as applying the features learned from an original problem to a new problem, often used when the new training dataset is too small [1]. Using a pretrained network (i.e. one that has been trained on a separate image database, for example, a natural image database such as ImageNet [2]), and then transfer-learning to a new dataset is a popular method in the computer vision field [3], [4]. Pretraining can also help to overcome the limited training data associated with the medical imaging field [5], for example in positron emission tomography (PET) imaging [6], arising from the associated processing and time costs [7].

Pretrained convolutional neural network (CNN) backbones have been useful for the task of transfer learning from natural two-dimensional images to medical images [8]–[10]. There are multiple established backbones available that have been pretrained on the ImageNet database, however, there is debate over the best performing model and different conclusions are drawn for different tasks. For example, the Inception [11], [12] family of architectures was the most used in medical imaging classification tasks [13]. Tamilarasi and Gopinathan [14] showed that using the InceptionV3 architecture outperformed VGG16 [15] for the task of binary classification of 200 magnetic resonance (MR) brain images into “abnormal” or “normal”, achieving an accuracy of 95.1% compared to 92.8%. The authors reasoned that the Inception architecture is wider (more and larger kernels) than and not as deep (fewer layers) as the VGG16 architecture and may have improved model performance on test data, as more feature maps were generated, hence, more image features were captured. However, other studies show that networks, such as VGG16 that are also used widely in the literature, may outperform Inception networks. For example, using a VGG16 architecture with pretraining via ImageNet achieved a 97.7% accuracy when classifying cytological images as papillary thyroid carcinoma or benign, compared to 92.8% for InceptionV3 [16]. In contrast to Tamilarasi and Gopinathan [14], Yadav and Jadhav [17] showed VGG16 pretrained via ImageNet, outperformed InceptionV3 when classifying chest x-rays into “Normal” or “Pneumonia present”. Other studies showed pretraining may not be required, for example, He *et al*. [18], suggest that pretraining may not be required at all, based on detection of the COCO dataset, with random initialisation of models perfoming no worse than using ImageNet pretraining. Both VGG16 and InceptionV3 backbones have also been used in histopathology image classification. It was shown that networks pretrained on natural images gave comparable performances to those trained with a large number (27,055) of histopathological images without pretraining [19].

Other studies have compared multiple established backbones for medical imaging tasks, specifically for classification tasks. For example, Rahaman *et al*. [20] compared 15 CNN backbones pretrained on ImageNet in the task of classifying 2D chest X-rays into “normal”, “pneumonia” or “COVID-19”. The authors used a total of 860 images for this task (a training:validation:testing split of 70:15:15 percent), and found that VGG19 [15] performed the best for this task [20]. This is in agreement with Naz *et al*. [21], who compared 11 different CNN backbones and froze only the first few layers to obtain general features. The task was classifying MRI brain images into “mild cognitive impairment vs. Alzheimer’s disease”, “Alzheimer’s disease vs. normal controls” and “mild cognitive impairment vs. normal controls”. The authors found that VGG19 [15] performed best for mild cognitive impairment vs. Alzheimer’s disease (99.27% accuracy), and VGG16 [15] performed best for the other two classifications (98.89% and 97.06% accuracy, respectively). Work by Shakhovska and Pukach [22] compared 5 different pretrained backbones for the task of diagnosing MRI knee images. This work found that different backbones worked best for different slice orientations and specific diagnoses. Similarly, Zebin and Rezvy [23] compared three different pretrained backbones (VGG16 [15], ResNet50 [24] and EfficientNetB0 [25]) for the task of classifying 2D chest X-rays into either “COVID-19” or “Pneumonia”. This study used these pretrained backbones as feature extractors, adding a new encoding layer, and found accuracies of 90%, 94.3% and 96.8% for VGG16, ResNet50 and EfficientNetB0, respectively. This was in contrast to Shakhovska and Pukach [22] who found that EfficientNet performed the worst for their task. However, in both studies, the number of backbones compared was limited.

Most research has been carried out in fields such as computed tomography (CT), MRI and microscopy [13], but there is very little work carried out on positron emission tomography (PET) [26]–[29] imaging and on image quality assessment tasks. However, clinical PET image quality assessment is important, especially in low-count PET reconstructions as this can cause a loss of resolution and increased noise [28], [30], [31], resulting in the image being unable to be used clinically. Low-count imaging may become an important tool to overcome radiation dose concerns. Reducing the radiation dose decreases the associated cancer risk for the patients [32], and the amount of radioligand needed, lowering the cost of manufacturing to Good Manufacturing Practice standards [33]. Automated evaluation of these low-count PET images alleviates organisational pressures on clinicians to read these images, and provides rapid assessments for large-scale reconstruction studies.

This work builds upon previous work by the authors [6], whereby an in-depth study into the impact of pretraining of the VGG16 backbone was carried out. The conclusion of Hopson *et al*. [6] was that including pretraining from a natural image database outperformed no pretraining for the task of automated PET image quality assessment. Retraining the last two convolutional blocks of the VGG16 backbone further improved model performance. This was in agreement with Singh *et al*. [34], who showed that for the task of detection of critical enteric feeding tube malposition in radiographs, pretraining the InceptionV3 architecture via the ImageNet database improved the area under the curve by 0.27 compared to training the same architecture without pretraining. These findings are also in agreement with Zhou *et al*. [35], who showed that unfreezing some of the weights of the InceptionV3 network (those after the “mixed 6” layer), improved model performance, achieving a maximum accuracy of 0.97.

The aim of this work was to provide a comprehensive comparison of the available CNN backbones pretrained via ImageNet for transfer learning from the natural image to the medical imaging field, specifically for the task of automated PET clinical quality assessment, for two-dimensional images. This will both target the paucity of literature for PET, and analyse multiple backbones, building on previous literature where only a few pretrained backbones were compared. Previous work [6] demonstrated that a balance between pretraining on ImageNet and re-training the architecture gave the best performance, thus, the impact of the level of pretraining for different backbones will also be investigated. This study aims to quantify what drives performance improvements of transfer-learned models from natural images to medical images, including model performance as a function of the number of trainable parameters, architecture and training regime.

## II Methods and Materials

### A Patient Dataset and Reconstruction

The same memory clinic patient dataset consisting of 13 patients was used as in Hopson *et al*. [6]. Each patient underwent both a PET-CT scan (acquired on a Discovery 710 PET-CT (General Electric, Chicago, USA)) and a PET-MR scan (acquired on a Biograph mMR simultaneous PET-MR (Siemens, Erlangen, Germany, field strength = 3T)), at the King’s College London & Guy’s and St Thomas’ PET Centre, as part of a wider study. The dataset was acquired by Dr Colm J. McGinnity and [18F]FDG was used as the radioligand in both the PET-CT and PET-MR scans. PET-MR scanning commenced 98±26 minutes post-injection of [18F]FDG. The MR images were acquired using a 3D T1-magnetisation-prepared gradient-echo (MP-RAGE) sequence for a duration of 30 minutes per patient. The patient demographics are shown in Table I.

**Fig. 1:**
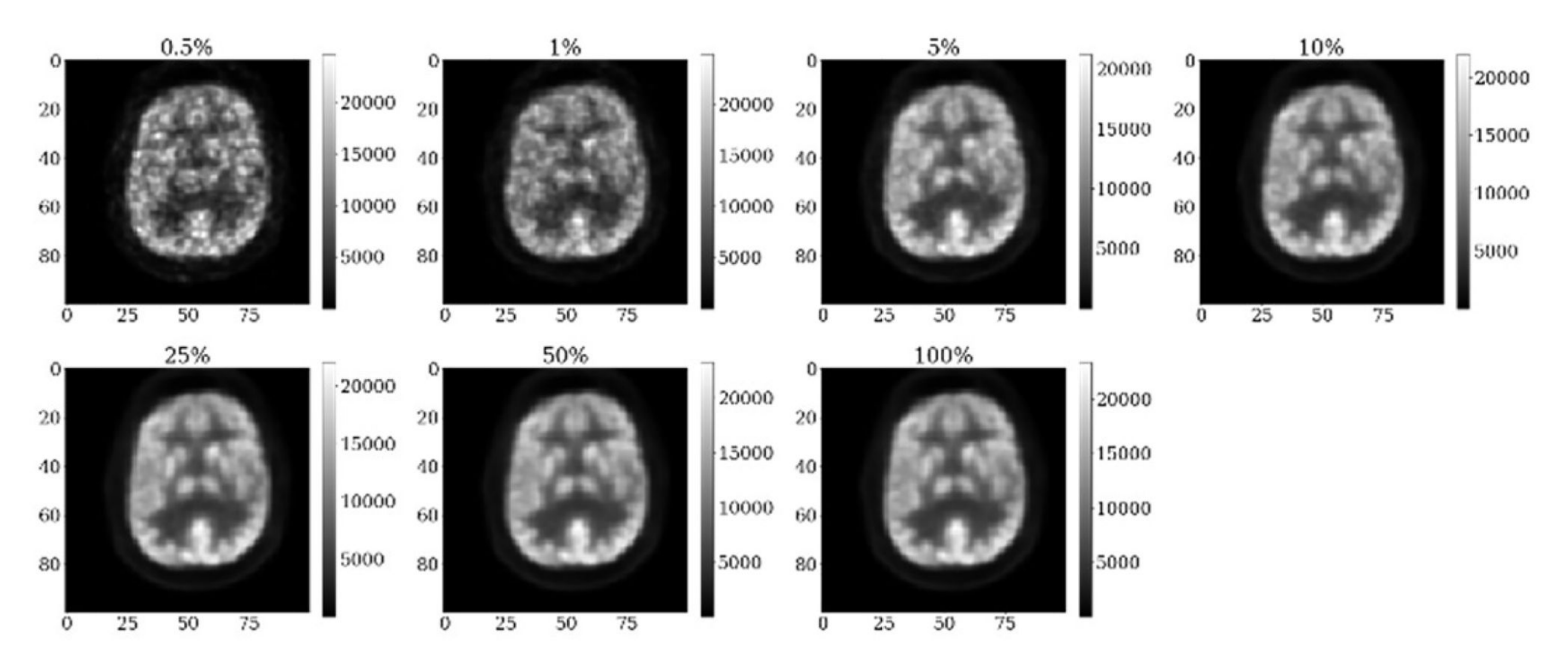
The same example cropped slice reconstructed with different percentages of counts, simulating 7 dose levels. The units of the values given in the colour bar are Bq/ml.

To reconstruct each patient dataset, Siemens e7 tools was used, using ordered subset expectation maximization (OSEM) with 2 iterations and 21 subsets to reconstruction into a matrix size of 344 × 344 × 127 voxels (voxel size of 2.09 × 2.09 × 2.09 mm^3^). The reconstructions has a 4mm post-reconstruction Gaussian filter applied, as per clinical standard. Each patient dataset was reconstructed at seven different count levels (0.5%, 1%, 5%, 10%, 25%, 50% and 100%), by resampling the list-mode PET data [36] (Fig. 1), simulating a range of lower injected doses administered to each patient. The clinicians were given 3 of the 7 count levels for each patient for clinical scoring (section II.C).

### B Preprocessing Protocol

Once reconstructed, all 3-D images were normalised between 0 and 1, to preserve the relationships among the original data values and bring all reconstructions to the same range prior to presentation to the network. The data were presented to the network as randomly extracted 2-D patches of size 80 × 80 to reduce the computational time of model training. Per reconstruction, 1000 patches were extracted from each of the sagittal, coronal and transverse planes, ensuring the whole of the 3-D volume was represented. A thresholding algorithm, inspired by [37] was applied, preventing the use of background-dominated patches. The average pixel value of both the patch and the whole image volumes were compared: if the average pixel value of the patch was <1/8^th^ of that of the whole image volume, then the patch was deemed as background and rejected. Within the initial 1000 patches per plane per reconstruction, ~200-500 were deemed non-background, thus 300 patches (100 per plane) per reconstruction were randomly chosen.

### C Clinical Evaluations

The whole image volumes were randomized and blinded upon presentation to 2 experienced clinicians. To reduce the time taken to carry out the clinical evaluations, the count levels were grouped into “low quality” (0.5% and 1%), “medium quality” (5% and 10%) and “high quality” (25%, 50% and 100%). A total of 13 patients at 3 different count levels (1 per group) were assessed by the 2 clinicians. This study could be classified as covering proof-of-concept evaluation and technical evaluation in the RELIANCE framework and used objective tasked-based clinical assessment [38], with the clinician defining the task on which the models were clinically evaluated. The clinicians scored each of the images based on 3 clinical quality scores designed by the authors [6]. These metrics were designed specifically by the authors in order to incorporate what the clinicians need to observe in a reconstruction to make a diagnosis. All three metrics have equal clinical relevance, as they all provide a different aspect of what is important about a reconstruction. These metrics were global quality rating (GQR), dominated by visual qualities such as sharpness and noise, pattern recognition (PR) used to judge whether any pathological patterns associated with memory problems were detectable, and diagnostic confidence (DC), used to determine if the image can be used to make an accurate diagnosis. These clinical metrics were scored as: 0 (“unacceptable”), 1 (“poor but useable”), 2 (“acceptable”) or 3 (“good/excellent”), with 0.5 scores allowed. The clinicians agreed within 0.5 (within label noise) for 91% of the images. A consensus reading session was carried out with both clinicians to discuss the images with the greatest discrepancies (i.e. ≥1). These agreed scores were then used (10 out of 117 total clinical scores); all other images used an average of the 2 clinicians’ scores (107 out of 117 total clinical scores).

### D Training the Deep Learning Backbones

**Fig. 2:**
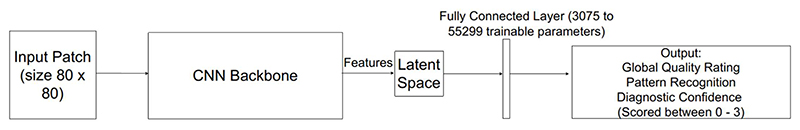
Schematic of the deep-learned network. A patch is inputted into the CNN backbone (Table II), which extracts features that lie in a low-dimensional space (latent space). A flatten layer is then used to change the dimensionality. The fully connected layer connects the latent space with the final output.

A total of 18 different established backbones were investigated (Table II). These backbones were chosen to ensure that 5 different architecture groups were investigated, and networks within in each group had with a different number of layers. For each of the models, the original encoding layers were replaced by a single flatten layer and fully connected layer. The encoding layers were changed to predict the 3 clinical metrics for this medical imaging task, instead of classifying natural images into 1000 different classes (summarised in Fig. 2). An Adam optimizer with a learning rate of 10^-4^ was used, with a batch size of 10 and a mean-squared-error loss function (given by Equation 1).

(1) $$MSE=\frac{1}{n}\sum_{i=1}^{n}{\left(y_{i}-{\hat{y}}_{i}\right)}^{2}$$

Where ŷ_i_ is the *i*th estimated value, y is the corresponding true value, and n is the number of samples.

Models were trained for 1000 epochs and saved only at the epoch where the validation loss was lowest. Initially, only the fully connected layer was retrained, with all weights initialised from ImageNet. The training dataset consisted of a maximum of 8 different patients at 3 dose levels each (24 reconstructions, equating to 7200 80 × 80 patches). A validation dataset consisting of two different patients at three dose levels each (total of six reconstructions, equating to 1800 patches of size 80 × 80) was used. Both cross-fold validation (running each model 6 times each with 2 different patients used for validation each time) and ensemble (running the same model 5 times with the same training and validation sets) methods were used (leave-one-out cross-fold validation). A further 3 unseen patients at 3 dose levels each (2700 patches) who were not included in the model training were used for testing.

### E Modifications to Training

The training protocol was adapted to investigate the impact of pretraining level. This was achieved by initializing the network with ImageNet weights and freezing the weights of the first 60% of network layers (Table III), in line with Hopson *et al*. [6], which found that unfreezing the last 2 blocks of a VGG16 backbone (i.e. the last 40% of layers) achieved the best performance on unseen data. The same training and validation sets as in section II.D were used, enabling a direct comparison.

### F Evaluation Metric and Implementation

Each model was tested on the same 3 patients at 3 dose levels each, with the mean-absolute-error (MAE) used for evaluation (given by Equation 2).

(2) $$MAE=\frac{1}{n}\sum_{i=1}^{n}\left|y_{i}-{\hat{y}}_{i}\right|$$

Where ŷ_i_ is the *i*th estimated value, y is the corresponding true value, and n is the number of samples.

Siemens e7 tools implemented in MATLAB (The Mathworks, Inc.) was used for reconstruction. All models were trained in TensorFlow [45] using the Keras [1] application programming interface. An NVIDIA Quadro RTX6000 24GB GPU or an NVIDIA DGX system was used.

## III Results

### A Comparison of Backbones as a Function of the Number of Trainable Parameters and Training Pairs

**Fig. 3:**
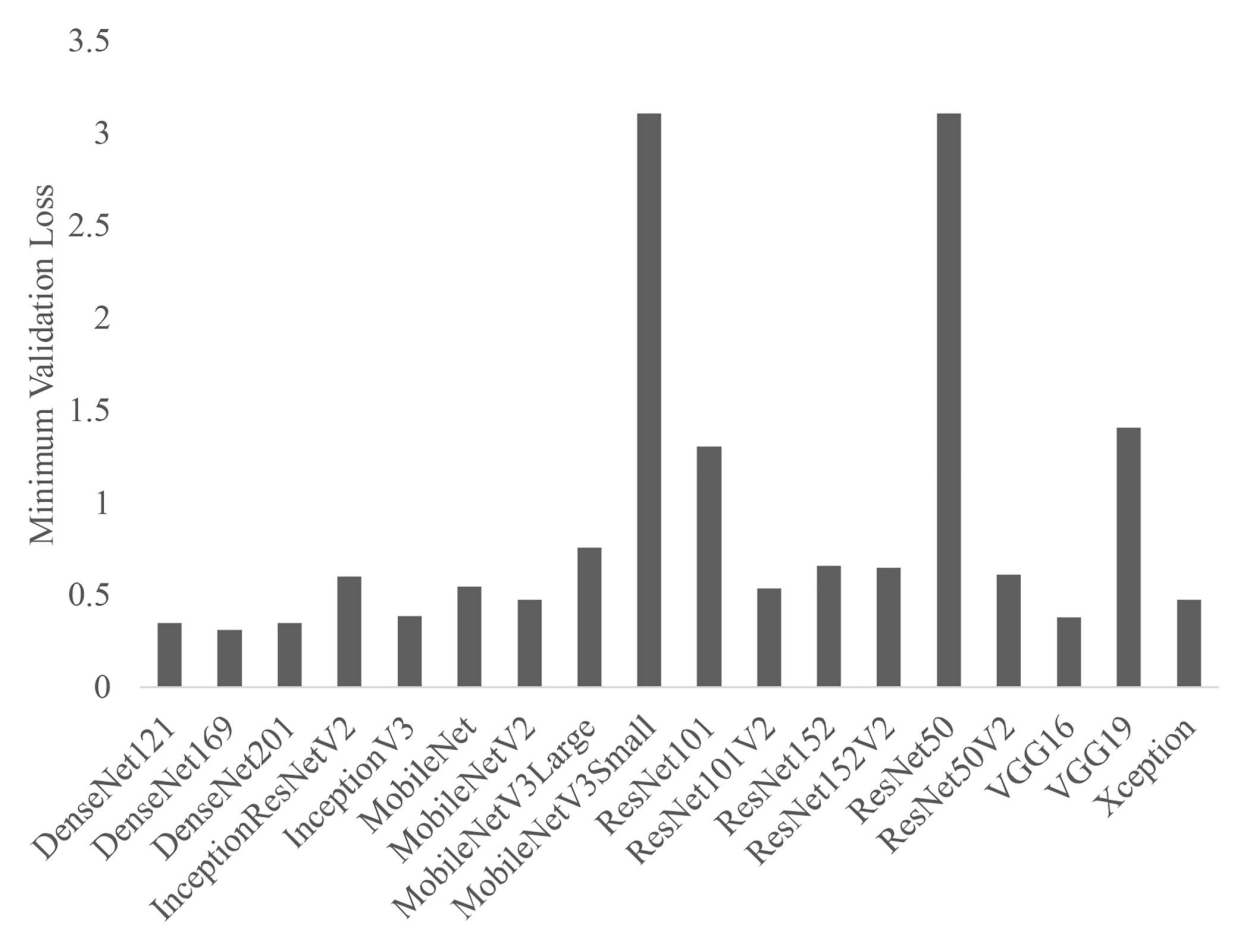
Minimum validation loss (mean-squared-error) of models. Pretrained weights were frozen and only the fully connected layer was trained.

**Fig. 4:**
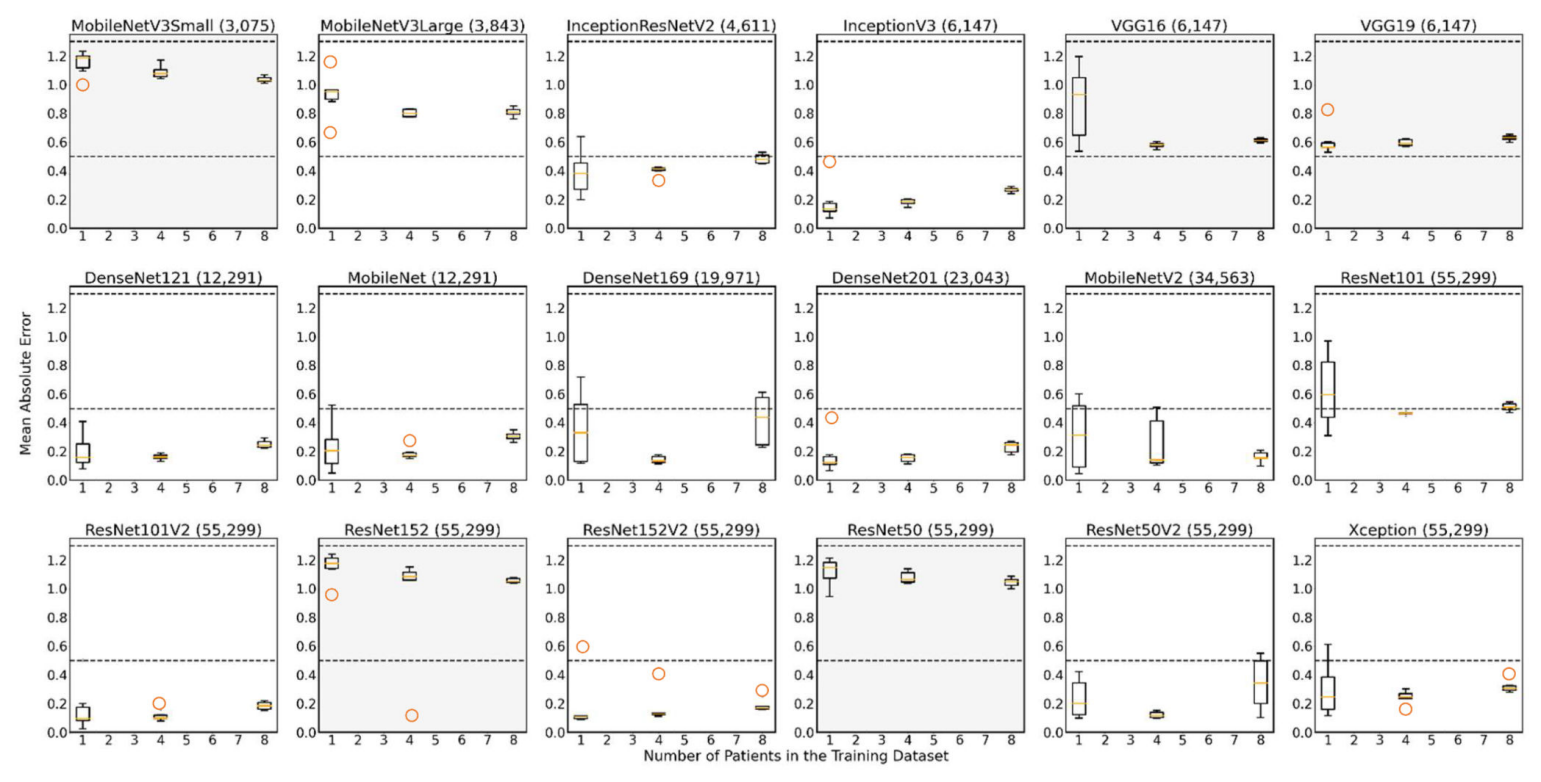
Training mean-absolute-error (MAE) at minimum validation loss for each of the architectures, training only the fully connected layer for *n* = 1, 4 and 8 patients in the training dataset, for 6 cross-folds. The brackets indicate the number of trainable parameters. Dashed lines: theoretical maximum MAE of 1.3 (random labels) and theoretical minimum MAE of 0.5 (label leniency). Orange line = median, outliers = circles. All models are ordered in terms of the number of trainable parameters, (fewest in top left, most in bottom right). Greyed plots = failed to train correctly, not used in Fig. 6.

For the 18 different architectures considered in this work (Table II), Fig. 3 shows the minimum validation loss during training when all pretrained ImageNet weights were frozen and only the final fully connected layer was trained with 8 patients. A minimum validation loss of ~ 0.5 (within label noise) was achieved for 14/18 backbones. Fig. 4 shows the impact of the number of training pairs on the training MAE for the 18 backbones. The boxplots were calculated over 6 cross-folds. The subplots have also been ordered to left-to-right, and top-to-bottom in terms of number of trainable parameters, when only training the fully connected layer, such that MobileNetV3Small (top-left) has the fewest trainable parameters, whereas from ResNet101 to Xception have the largest number of trainable parameters (bottom-right).

For each backbone, the boxplots for training error at the point of lowest validation loss are shown for *n =* 1, 4 and 8 patients in the training dataset. The x-axis represents the number of patients from which the patches were extracted for training. For example, *n =* 1 was equivalent to 900 individual patches, and *n=*8 was equivalent to 7200 individual patches. If the model failed to train (i.e. did not fit to the training data), then the MAE oscillated close to the theoretical maximum (~1.3) (random labels). If the model succeeded in training, the error reduced close to 0, regardless of the number of patients in the training dataset. For example, the MAE for ResNet152 consistently oscillated around 1.3, compared to below 0.5 for e.g. DenseNet121. ResNetV2s had a training error close to 0, whereas the ResNetV1s failed to train (except for ResNet101).

**Fig. 5:**
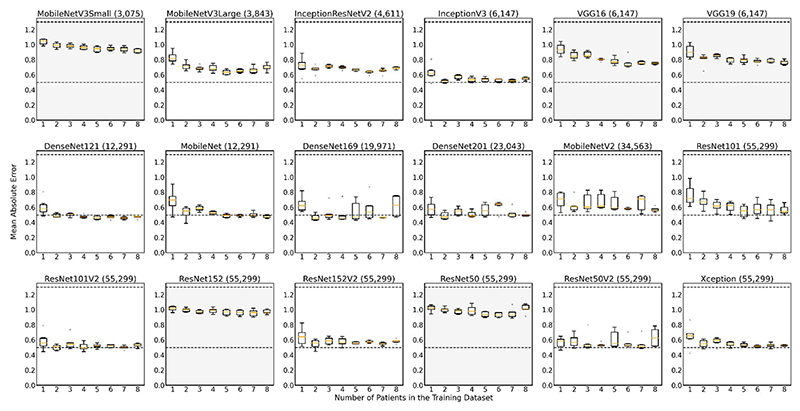
The impact of the amount of training data (up to and including 8 patients) on the generalisation (in terms of mean-absolute-error (MAE)) to unseen test data, over the same 6 cross-folds as used in Fig. 4. Only the fully connected layer was trained. The model backbone name is given above each plot, with the brackets indicating the number of trainable parameters in each model. Dashed lines: theoretical maximum MAE of 1.3 (random labels) and theoretical minimum MAE of 0.5 (label leniency). Orange line = median, outliers = circles. All models are ordered in terms of the number of trainable parameters, (fewest in top left, most in bottom right). Greyed plots = failed to train correctly, not used in Fig. 6.

Fig. 5 shows the impact of the amount of training data (up to and including 8 patients) on the generalisation to unseen test data, over the same 6 cross-folds as used in Fig. 4. A decreasing MAE with more data in the training dataset was expected. For the majority of the architectures, the MAE did decrease with an increasing number of training pairs. There were a few exceptions, for example, ResNet152 and ResNet50, whereby the test MAE tended to oscillate around the theoretical maximum MAE of 1.3, consistent with their failure to train (Fig. 4). The graphs were ordered by the number of trainable parameters in the network, with the fewest in the top left, and most in the bottom right. Generally, for all sizes of the training dataset, the MAE tended to decrease between InceptionV3 and DenseNet169, corresponding to a number of trainable parameters of 6,147 to 19,971, with the exception of Xception. By comparing Figs. 4 and 5 those models that were able to train (low training MAE) resulted in a better test MAE and had a much smaller interquartile range than those that did not (Fig. 5).

**Fig. 6:**
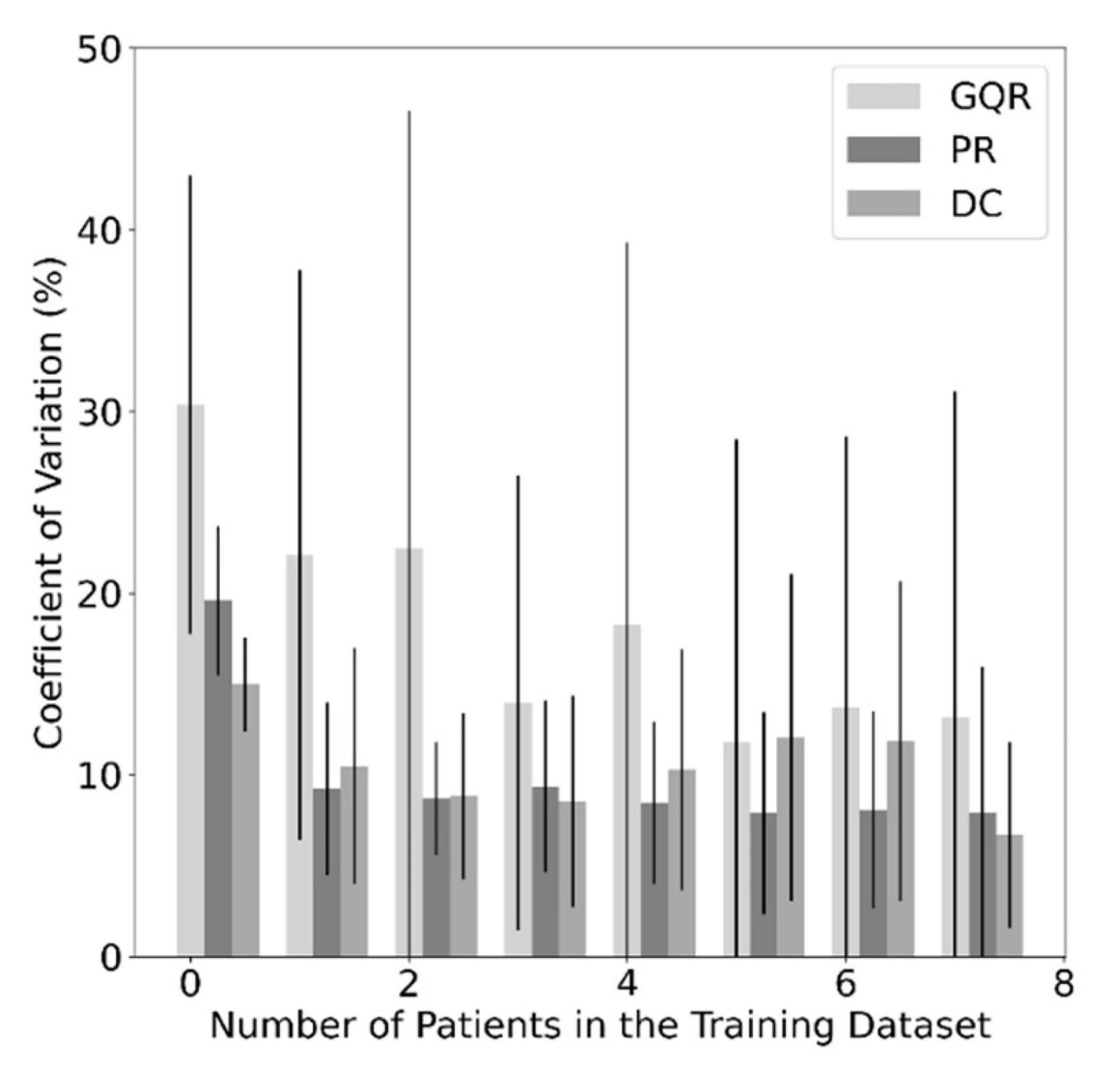
Coefficient of variation (CoV) of the predicted quality metrics (global quality rating (GQR), pattern recognition (PR) and diagnostic confidence (DC)) averaged over all backbones as a function of the number of patients in the training dataset. Those with a CoV of 0 were omitted (see section III.A).

The CoV measured the spread in the predictions for each metric (Fig. 6). A CoV of 0 for MobileNetV3Small, VGG16, VGG19, ResNet152, and ResNet50, indicated a failure to train for all or some metrics (grey in Figs. 4 and 5); these models were omitted from Fig. 6. As the number of patients in the training dataset increased, the CoV decreased for all metrics.

**Fig. 7:**
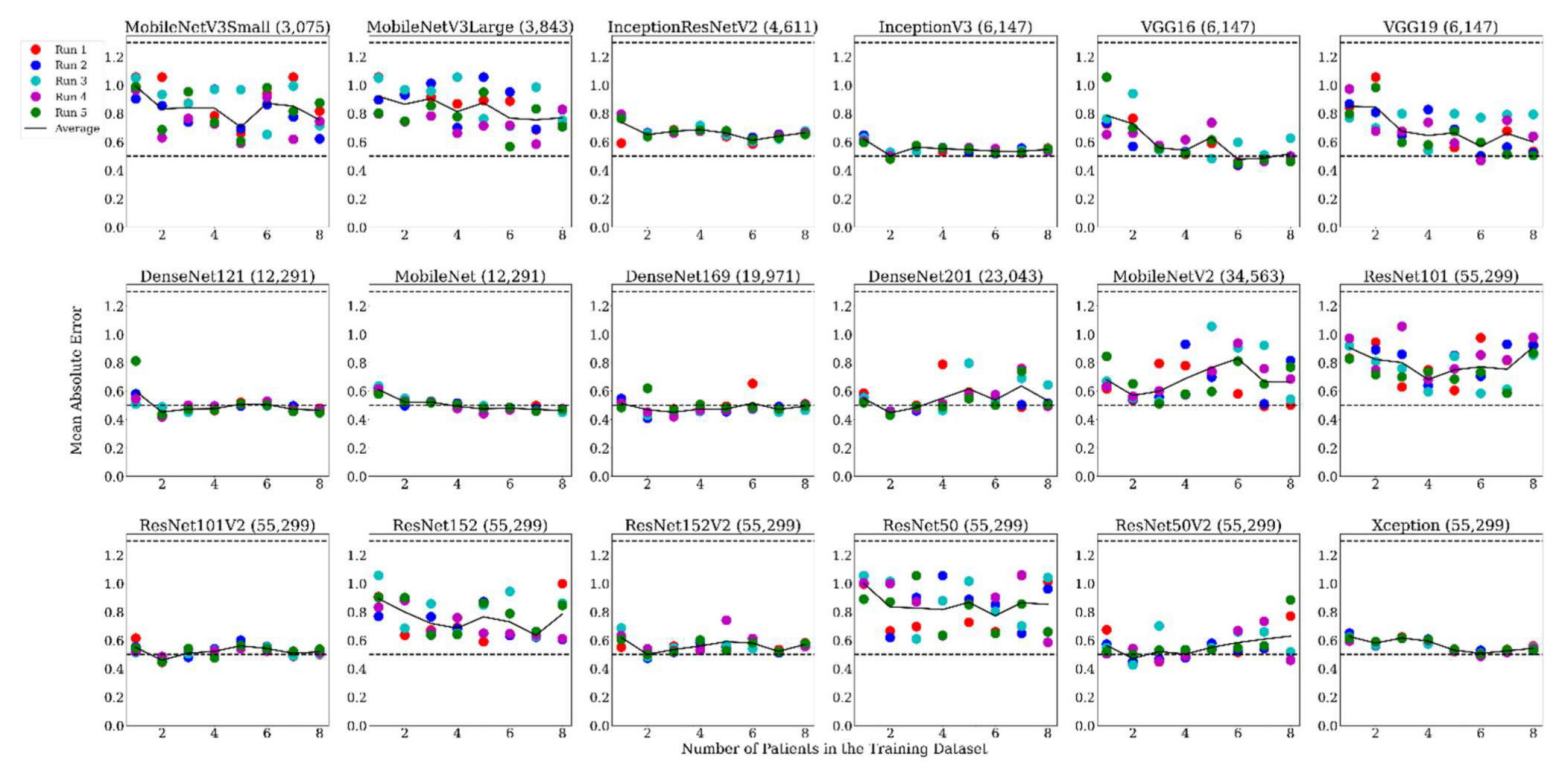
The mean-absolute-error (MAE) for an ensemble of retrained networks (retrained 5 times) for unseen test data at minimum validation loss for each architecture, as a function of the number of patients in the training dataset. Dashed lines: theoretical maximum and minimum MAE. Coloured sets: different training runs. Solid line: average of the 5 training runs.

A single cross fold training was run 5 times (Fig. 7) for further validation. Similarly to Fig. 5, the MAE decreased with more patients, for models between VGG16 to DenseNet169 (6147 to 19971 trainable parameters), where the MAE consistently fell below 0.5 i.e. within label noise. The error was more erratic outside this range, again with the exception of Xception.

### B Training as a Function of Frozen Weights

**Fig. 8:**
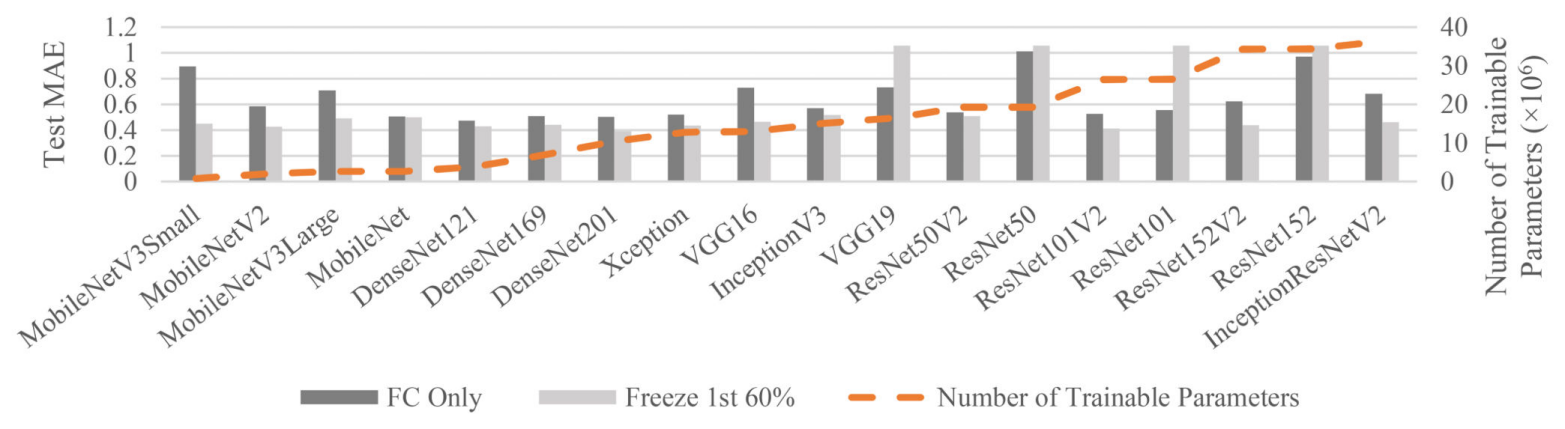
Mean-absolute-error (MAE) for test data at minimum validation loss for each architecture, when freezing the weights of all layers except the fully connected (FC) layer (dark grey) or of the 1^st^ 60% of layers (light grey). Orange dashed line = number of trainable parameters when unfreezing the weights of the last 40% of layers.

Fig. 8 shows the impact of freezing only the pretrained weights of the first 60% of layers for a single cross-fold. Apart from 4/18 backbones tested (VGG19, ResNetV1s), freezing the weights of the first 60% of layers improved the MAE to ≤0.5, compared to 7 when training only the fully connected layer. MobileNetV3Small showed the largest improvement (from 0.89 to 0.44).

## IV Discussion

Each backbone was initially evaluated on a validation dataset consisting of two patients. With the exception of MobileNetV3Small, ResNet101, ResNet50 and VGG19, all other backbones achieved a minimum validation loss of ~0.5 (Fig. 3). Later versions of architectures performed better on the validation dataset than their earlier counterparts. Increasing the number of patients in the training dataset, caused the test MAE to decrease (Fig. 4). This was the case for most architectures, but was most clearly shown for VGG16 and MobileNet. A larger dataset can improve model performance because it reduces the risk of overfitting to the training dataset [46]. For VGG16, Fig. 4 is in line with Hopson *et al*. [6], which showed that only training the fully connected layer of the VGG16 backbone network failed to generalise well to unseen data, which was due to the model failing to fit to the training data.

The CoV for each metric, and so the variation in the predictions, decreased with more patients (Fig. 6), in line with Sharma and Mehra [47], who showed larger training datasets improved model robustness.

When only training the fully connected layer, the model performance was best in terms of MAE between approximately 6,000 to 20,000 trainable parameters (Fig. 5). Some backbones, such as DenseNet201, had an increase in MAE with an increasing number of patients in the training dataset. This was perhaps because the model did not have enough capacity to fit to an increased amount of training data. However, this may also be due to random variation in the multiple training runs, as there was no difference in the training errors between different numbers of patients, i.e. the standard deviations overlapped. The worst performing models were outside of this range, creating a “U-shape”, with ResNet152 and ResNet50 having the highest MAEs. The only exceptions were for the ResNetV2s and Xception. This high MAE can be explained by the training error (Fig. 4). For these models, the training error oscillated around the theoretical maximum MAE of ~1.3, i.e. they failed to fit to the training data, meaning that the resulting test error also oscillated around this MAE. For all six cross-folds, the training and test error still oscillated around the theoretical maximum MAE of ~1.3. Conversely, for the models with 6,000 to 20,000 trainable parameters, the training MAE was consistently ≤ 0.5 (Fig. 4), within the leniency for the labels, suggesting an optimal number of trainable parameters to achieve the best model performance for this task. This was perhaps because with fewer trainable parameters, the model did not have capacity to generalise well to the unseen data. Classically, however, too many trainable parameters can cause overfitting to the training data [48], and generalisation issues [49], to unseen test data.

A similar trend was shown when using ensembles (Fig. 7), with the variation in each trained backbone arising from stochastic optimisation and the varied presentation order of the data. Generally, with more patient in the training dataset, the lower the test MAE. Similarly to Fig. 5, a “U-shape” curve was shown as a function of the number of trainable parameters, supporting the idea that too few or too many trainable parameters may be detrimental to the model performance at test-time. Thus, it could be concluded from this result that when using established networks and only modifying the encoding layers, trainable parameters are one of the driving forces that improve performance at test-time. The lower MAE achieved by the ResNetV2 architectures compared to their corresponding V1s (Figs. 5 and 7) may be explained by the fact that these architectures were designed to overcome the problem of model performance degradation with increasing model depth [24]. Compared to ResNetV1s, ResNetV2s remove the last non-linearity from the residual unit, creating a direct path between the input and output as an identity mapping, improving the accuracy of the model [43]: model performance should not degrade with increasing depth, improving generalization to unseen data. This explains [39] the lower MAE achieved by the DenseNets, as they were designed such that the feature maps from all preceding layers were the inputs into subsequent layers. Xception also makes use of residual connections and removes intermediate activation functions. Thus, backbones with residual connections and that remove non-linearity functions are important for use as feature extractors.

However, one limitation of this study is that perhaps there may be some overlap in the appearance of some patches, as they were extracted from similar regions, which may cause some overfitting of the model to the training data. Additionally, it would be preferable to have greater than 13 independent patients, to draw more robust conclusions, but this study may be used as a proof-of-concept study. Nevertheless, this number of patients is comparable to other studies, such as Corda *et al*. [50].

By pretraining on ImageNet but unfreezing the weights of the last 40% of layers, the MAE at test-time decreased for 14/18 architectures (Fig. 8). This was in agreement with Hopson *et al*. which used the VGG16 backbone [6], but this study also showed it held true for other backbones. Unfreezing some weights achieved a balance between using ImageNet-specific features, whilst also maintaining the benefit of pretraining on a large number of images [6], [51], [21]. We speculate that whilst very numerous, the number of possible smaller-scale lower-level features (such as noise textures) is ultimately relatively limited for images coming from any domain, and therefore these low-level features (captured by the early layers of the CNN) are generally shared by both natural and medical images. The relative amounts of these small scale features differ between natural images and PET images, but detection of these small common features can be learned even by considering only natural images. It is only the later layers of the CNN encoder that need to be different to accommodate the different broader level feature differences of brain PET images, and therefore, this could be why we found that unfreezing and fine tuning these later layers gave performance improvements. The models were then in the “modern” regime [52] [49] (training data was memorised [53]), as the maximum number of samples (7200) was much lower than the number of trainable parameters. In the “classical” regime, the training data is not memorised [54]. Thus, the “modern” regime may improve model performance.

## V Conclusion

Multiple backbones can be used for transfer-learning from a natural image database to a small medical imaging dataset. When pretraining via ImageNet and freezing all weights, models with ~6000-20000 trainable parameters achieved the lowest MAE. When unfreezing the weights of the last 40% of layers, model performance improved for 14/18 architectures. Models with residual units (e.g. DenseNets and ResNetV2s), were suited to this task, as was the over-parameterized regime. Future work may include applying these initial findings to other similar tasks, to determine their generalisability, and potentially comparing to handcrafted features. Future work may also cross into explainable artificial intelligence [55]–[57], whereby the interpretation of the images by the model could be explored.

## Figures and Tables

**Table I:** Patient Demographics

Patient Number	Sex	Age	Weight (kg)	Injected FDG Dose (MBq)
1	Female	61	64	221.71
3	Female	64	58	240.00
5	Female	71	51	213.18
6	Male	68	100	234.8
10	Male	51	74	244.37
17	Male	58	83	189.76
27	Female	51	77	242.72
32	Male	62	92	229.15
37	Male	46	91	233.41
48	Female	58	94	212.82
54	Male	61	103	246.00
60	Male	43	77	234.34
64	Male	77	80	228.75

**Table II:** Trainable Parameters when only the Fully connected Layer was Trainable

Model Name	# Trainable Parameters
DenseNet121 [39]	12,291
DenseNet169 [39]	19,971
DenseNet201 [39]	23,043
InceptionResNetV2 [12]	4,611
InceptionV3 [11]	6,147
MobileNet [40]	12,291
MobileNetV2 [41]	34,563
MobileNetV3Large [42]	3,843
MobileNetV3Small [42]	3,075
ResNet101 [24]	55,299
ResNet101V2 [43]	55,299
ResNet152 [24]	55,299
ResNet152V2 [43]	55,299
ResNet50 [24]	55,299
ResNet50V2 [43]	55,299
VGG16 [15]	6,147
VGG19 [15]	6,147
Xception [44]	55,299

**Table III:** Trainable Parameters After Freezing the Weights of the First 60% of Layers

Model Name	# Trainable Parameters
DenseNet121 [39]	3,811,075
DenseNet169 [39]	6,958,019
DenseNet201 [39]	10,425,859
InceptionResNetV2 [12]	36,295,171
InceptionV3 [11]	15,106,307
MobileNet [40]	2,675,715
MobileNetV2 [41]	1,993,731
MobileNetV3Large [42]	2,645,491
MobileNetV3Small [42]	770,755
ResNet101 [24]	26,484,227
ResNet101V2 [43]	26,471,939
ResNet152 [24]	34,314,755
ResNet152V2 [43]	34,298,883
ResNet50 [24]	19,243,267
ResNet50V2 [43]	19,234,307
VGG16 [15]	12,985,347
VGG19 [15]	16,524,803
Xception [44]	12,761,595

## Data Availability

the patient data supporting this article can only be shared in anonymized form due to the type of participant consent obtained; please contact the corresponding author if required.
